# Concealed coexistence: Reproductive choice and coercion in Timor‐Leste

**DOI:** 10.1111/maq.70056

**Published:** 2026-01-24

**Authors:** Laura Burke

**Affiliations:** ^1^ Institute for Social Anthropology Austrian Academy of Sciences Vienna Austria

**Keywords:** coercion, choice, family planning, reproductive rights, Timor‐Leste

## Abstract

Choice is a central concept in reproductive rights. However, a discourse of choice in reproductive health can also mask precisely the act it aims to protect against: coercion. Whilst choice has been explored extensively in studies of reproductive rights and justice, understandings of coercion are fragmented and under‐theorized. This article explores the relationship between coercion and choice, not as a binary but as a coexistence in which they overlay and conceal one another. Drawing on ethnographic research amongst health professionals during family planning training in Timor‐Leste, this article shows how a discourse of choice obscures coercive structures and practices, whilst coercive approaches can paradoxically reveal hidden choices. I argue that this coexistence, characterized by concealment, leads to iatrogenesis—medical harm with immediate and lingering effects. By recognizing the coexistence of choice and coercion, and revealing their concealment of one another, we might limit iatrogenesis and enable greater reproductive freedom.

## INTRODUCTION

“We won't force you…but you can use these methods to space your children and give them more love”, smiled Rita^1^ a midwife in Timor‐Leste's central mountains. Rita was advising a woman at the local clinic as part of her practical training in family planning. Before the client and her husband entered the room, Rita's supervisor reminded her that she had not yet practiced an IUD insertion. Rita started the consultation by explaining the benefits of family planning. She then named the methods by pointing to the pictures on her WHO chart. She described the condom—“the man wears this”; the Billings method—“you count the days”; the pill—“you must take it every day”; the copper IUD—“you put it in your womb”. Rita paused on the IUD, “this one is really good, it doesn't have side effects, and when you remove it, you can get pregnant again”. Glossing over the other methods, Rita focused on the IUD and its benefits: “It has no side effects and no “medicine”, no hormones, and you will still have a period every month”.^2^

The couple were asked which method they would like to choose. The client's husband gestured towards the IUD. The client, cradling her 2‐month‐old, looked at her husband, then nodded., Smiling brightly Rita said “if you want you can take it out at any time…we are not *forcing* you, we are just giving you information.” Despite Rita using a language of “choice”, she was persuading the client to choose the IUD. The client nodded in acceptance.

The insertion of the IUD was painful. The client was still healing from labor and It took some time for Rita to find the cervix as. She called for help from the training facilitator. When the client tensed with pain she was sternly told to relax, and her legs were roughly put into stirrups. The facilitator gave no reassurances when she inserted various implements. After a painfully long and uncomfortable time for the client, the procedure was complete. The client was then ushered out as the bed was needed for an emergency.

Coercion had been denounced from the beginning of the consultation, and “choice” was emphasized. Yet Rita had been instructed to find an IUD patient to practice on, and the client had been persuaded. The client was then treated badly and subjected to a painful experience which could be considered obstetric violence.

How did this happen? How do choice and coercion coexist in family planning consultations? Drawing on ethnographic research with medical practitioners in Timor‐Leste, I examine the intimate relationship between choice and coercion. Definitions of choice and coercion differ across time, space and disciplinary literature; broadly, however, here coercion refers to actions that make someone do something against their will, or without their knowledge, and choice refers to a person's ability to choose something autonomously. The concept of reproductive “choice”, or a “right to choose”, is a central pillar of global reproductive health. In a time when reproductive rights are becoming increasingly restricted, the concepts of choice, autonomy and agency are at the forefront of public feminist discourse. To understand these restrictions on rights, there have been calls to return to an analysis of coercion, which has been much less discussed (Boydell et al., 2023; Senderowicz, 2019). This paper contributes to this conversation, suggesting that to understand coercion, and choice, we must examine the relationship between them not as binary categories but as coexistent, and pay attention to how they oscillate, overlay and conceal each other in discourse and practice.

A “both” rather than an “either‐or” approach shows how choice and coercion coexist unstably in a range of “immediate and lingering presences” (Varley & Varma, 2021, p. 143). Identifying and categorizing different forms of choice or coercion along a spectrum is useful in practice, yet it risks losing the nuances of how coercion changes over time and space (Boydell et al., 2023). By theorizing the relationship as one of concealment, I show how choice and coercion interact with one another, obscuring and overlaying categorizations, leading to a broad range of iatrogenic harm.

My approach makes visible the hidden harms and discourses that limit reproductive freedom in very unequal contexts. Presenting data from a family planning training program in Timor‐Leste, this article shows how: a) “choice” is presented as freedom from coercion, b) how a discourse of choice conceals coercive practices and structures that impede choices, and c) how a coercive approach reveals a previously obscured but desired choice. First, I give an overview of the concepts of choice and coercion in reproductive health, rights and justice.

## CONCEALED COEXISTENCE

“Choice can change the world”: the United Nations Population Fund's report *The Power of Choice* describes how “choice” improves the well‐being of women and girls, transforming families, societies and global development (UNFPA, 2018). The uncomfortable truth is that this language of choice in family planning programs was the result of protesting coercive practices of population control.

In the mid‐20th century, concerns over population growth led states to adopt family planning policies that prioritized population control over individual rights. These policies often compromised women's bodily autonomy, resulting in coercive practices worldwide: sterilizations of Black women in the U.S. (Ross, 1992), forced abortions in China (Greenhalgh, 1994), abortion bans and body policing in Romania (Kligman, 1998), and sterilization incentives in famine‐affected areas of Bangladesh (Hartmann, 1985). Rooted in eugenicist ideology, these efforts were frequently backed by global north institutions advancing population agendas (Connelly, 2008).

On the global scale, feminist movements were calling for a move away from population policies and the top‐down family planning implementation which led to coercion and abuse. In the US, feminist activists used the framework of reproductive and women's rights to campaign for access to contraceptives and abortion. Roe v. Wade (1973) legalized abortion, focusing on concerns with choice over conception and motherhood (Ross & Solinger, 2017). Under pressure to appear nonconfrontational, proponents adopted the term “choice”, which glossed over less accepted language such as “abortion” (Solinger, 2001). “Choice” thus began to replace the language of “rights” (Solinger, 2001).

Simultaneously, a “reproductive health” approach to family planning was developed by the Ford Foundation, International Women's Health Coalition, Population Council, and WHO as a response to the limitations and abuses of earlier population policies (Lane, 1994). This rights‐based framework centered on women's health and emphasized voluntary, non‐coercive implementation. The 1994 Cairo International Conference on Population and Development (ICPD) embraced this approach, though some viewed it as another form of population control (Lane, 1994). Senderowicz (2019) notes that “coercion” was rejected at least 13 times in the ICPD Program of Action, without being defined. As such, the reproductive health model was grounded in negative freedom—freedom from coercion—rather than a proactive commitment to autonomy and failed to specify what forms of coercion it aimed to avoid or mitigate.

As the language of choice dominated abortion politics in the U.S., it failed to capture the reproductive inequalities experienced by Black and Indigenous groups (Solinger, 2001). Building on the reproductive rights framework, activists and scholars used reproductive *justice* to critique the “choice” discourse, arguing that systemic racism, classism, and sexism create unequal conditions that limit real choice (Ross & Solinger, 2017). As the main principle of women's reproductive freedom, “choice” is a politically compelling idea, but it is insufficient and problematic (Petchesky, 1980). It aligns with liberal democratic values and appeals to notions of equality, empowerment and freedom. However, in pursuing these liberal ideals, “a woman's right to choose” evades the details of when, under what conditions, and for what purposes, choices can *actually* be made (Petchesky, 1980, p. 669). Given the shaky history of how choice came to stand for reproductive rights, with obliviousness of race, class and privilege, choice is a “remarkably unstable and undependable foundation” for bodily autonomy (Solinger, 2001).

We must ask how useful the rhetoric of choice is, what it has become and who it is for. Gaard (2010) argues that feminists have lost control of the word “choice”, which has been repurposed and sold back to them as consumers of fertility treatments. In many places, having or not having children is now seen as a choice, yet when entrenched and intersecting inequalities exist across the life course, choice is little more than a “fantasy” (Crist, 2020: ibid). Choice “masks” and “disguises” unequal reproductive environments (Ross & Solinger, 2017, p. 47). The discourse of choice thus creates the possibility of equality, whilst simultaneously masking inequalities.

Agency, or a capacity to act, has been a key concept for gender scholars (see Unnithan, 2004). However, in the reproductive rights discourse agency is often conflated with individual choice or gender empowerment, assumed as something that is either present or lacking, rather than something negotiated and dynamic. From a western reproductive rights perspective freedom to engage in sexual intercourse may be read as an expression of agency. However, when reproduction is viewed as a wider process beyond a singular event e.g. sexual intercourse or pregnancy, there are also simultaneously experiences of constraint and control, rather than only agency and assertion (Butt & Munro, 2007). Attention to reproduction as a process shaped by unequally distributed power relations can reveal the different forms of power and agency that coexist (Burke, 2022b; Murphy, 2017). Here I draw attention to how different actors use a discourse of choice *and* various forms of coercion, which coexist in the same space and act upon one another.

### Relocating coercion

Concepts such as reproductive rights or reproductive justice are developed in a particular activist context, but they can be re‐interpreted and used for projects with aims that differ from the initial agendas they were intended for—and they are sometimes entirely co‐opted by groups following eugenicist ideas (Sasser, 2018). In development agendas, choice has gone beyond being about individual rights, rather it is a tool for “realizing all the sustainable development goals. THAT IS THE POWER OF CHOICE” (UNFPA, 2018, p. 1). Choice has become a celebrated ideal in healthcare. As a liberal value, individual choices are part of idealized modern democracy in contrast to restrictions by community and tradition (Mol, 2008, p. 3). A “choice” rhetoric thus acts as a promise of modernity and progress. Here family planning is equated with national development which is economically and morally incentivized (Murphy, 2017).

Early skepticism that “reproductive health” simply replaces population policies with a new language was not unfounded. Practices of population control, and advocacy for it, continue today under the guise of social and environmental justice (Sasser, 2018), human rights and women's empowerment (Bhatia et al., 2020). The use of the term “voluntary” itself requires critical analysis, as current family planning programs might reject population control but still target populations with marketing campaigns to increase contraceptive use (Hendrixson, 2019; Nandagiri, 2021). The discourse of choice becomes an illusion, masking the power structures of coercion through omission, obligation, shame, and pressure. Coercion in some family planning programs has been hidden in plain sight, in part due to a lack of consensus on language and categorization across disciplines (Boydell et al., 2023).

The focus on choice in reproductive matters has left coercion in its shadow. There is the risk, that by addressing negative elements in the reproductive rights domain we fuel the fires of those who seriously seek to limit reproductive freedom (Murphy, 2010). However, it is only through unmasking power hierarchies that we can transform them and advance sexual and reproductive health and rights (Schaaf et al., 2021). Little attention has been paid to coercion and what it entails (Senderowicz, 2019). There have been longstanding silences and inaction around coercion in some programs. Long‐acting forms of contraception (LARC) such as implants and IUDs are associated with coercion across literature in social science, law, and public health and medicine (Boydell et al., 2023). However, each discipline classifies coercion and assigns responsibility differently, providing only a partial picture (ibid).

A “choice” discourse born from a movement *against* coercion, has left critical silences around coercive practices. Coercive practices range from the overt (intimidation and force) to the subtle (bias counselling and persuasion) to free, full and informed choice. Choice and coercion are thus not binary oppositions (Nandagiri, 2021) and viewing them as such can harmfully gloss over the nuances of individual experiences and unequal contexts. Instead, we might view coercive practices as a spectrum of behaviors (Senderowicz, 2019). This is undoubtedly helpful for practitioners to categorize the different forms coercion can take. Additionally exploring how coercion relates to choice can show where these concepts also *intersect*. Unnithan (2022) shows how India's family planning policies promote rights‐based approaches to family planning whilst simultaneously returning to more coercive efforts of reproductive control. She suggests there may be a strategic policy rationale behind including both conflicting approaches in policies. The state is caught between liberalism and developmentalism and thus rights‐based approaches and coercion co‐exist in policy so the state can *show* care for citizens whilst still governing reproduction.

Examining these conflicting concepts of choice and coercion, I explore their coexistence in family planning practice in Timor‐Leste. I show how they coexist, overlaying one another in a relationship characterized by concealment. The Indonesian occupation of Timor‐Leste (1975–1999) resulted in the deaths of approximately one‐third of the population. Amid the human rights abuses committed by the Indonesian military were reports of forced sterilization, coerced contraceptive use, and the covert injection of women with contraceptive drugs (Sissons, 1997). Popular allegations suggested that the Indonesian family planning program (I, *Keluarga Berencana: KB*) was implemented with genocidal intent, however, there is little public conversation about this aspect of the Indonesian occupation, particularly within the current reproductive rights approach to family planning.

This account shows how choice is presented as freedom from coercion whilst the history of coercion itself is shrouded in silence. In the section below I explore conceptualizations of choice and coercion during a ten‐day family planning training course for medical professionals at Lauhili^3^ clinic near Maubisse, a small town in Timor‐Leste's central mountains. Maubisse was my primary field site during 18 months of ethnographic fieldwork (2018–2019), on the topic of reproduction in post‐conflict Timor‐Leste. Since regaining independence in 2002, Timor‐Leste experienced rapid population growth and the world's highest fertility rate (Saikia et al, 2009). My research investigated how communities were reproducing life in the wake of conflict and crisis through ethnography at a network of health centers with health care workers and volunteers, civil society groups and customary and religious leaders (Burke, 2022b).^4^ The family planning training was part of a program to improve child and maternal health in the municipality of Ainaro, which had the highest indicators of maternal and infant mortality in the country. I knew many of the participants as health professionals from local clinics and they consented to my participation as a trainee and a researcher^5^. I introduce Timor‐Leste's reproductive politics through an account of the first days of the training at Lauhili clinic.

## CHOICE AS FREEDOM FROM COERCION IN TIMOR‐LESTE

In independent Timor‐Leste, development narratives champion reproductive rights for national prosperity. However, these clash with conservative Catholic and nationalist leaders who fear promiscuity, a degradation of morals and tradition, and diminishment of the population. There are mixed attitudes about contraception in the Catholic Church in Timor‐Leste, but it plays a significant role in reproductive health and rights (Richards, 2015). My interlocutors expressed fears that the Church was always “watching”. Health campaigns risked intervention if they publicly promoted contraceptives. The main health center in Maubisse was partnered with the Church and did not provide condoms or hormonal contraception.

The family planning training afforded a rare moment in my fieldwork where actors from all areas of the family planning sector came together, and where “choice” was *taught*, and discussed. It involved local, national and international NGOs; the state and religious institutions; community doctors, nurses and medical and policy specialists, as well as patients or “clients”.

In his opening address to a room of participants, facilitators and funding representatives, the Director of the National Health Institute emphasized:

“…you mustn't force anyone to use family planning (KB)*…*they will choose for themselves, you can't force them. You are not sitting this training to then make people use it. It is the right of people, of the family, to choose what they want to use, and you are to share the information.”

The Director used the Indonesian acronym “KB” (*keluarga berencana)* to refer to family planning as opposed to the Tetum, *planeumentu familia*. This choice of words, alongside his positioning of the right to choose in relation to noncoercion, speaks directly to the history of family planning in Timor‐Leste. The association of the Indonesian KB program with genocide, against the Timorese population was raised by several of my interlocutors. One described how Timorese resisted the program and reappropriated the acronym KB to mean they were practicing “*Keluarga Besar”*, Big Families, rather than *“Keluarga Berencana*”, Family Planning.

During the occupation sexual violence against women was used as a form of intimidation and torture (Coomaraswamy, 1999). Public testimony addressed coercion in the KB program, and the covert sterilizations of young girls at a National Public Hearing during the Peace and Reconciliation process (CAVR).^6^ Reports also document the coercive administration of contraceptives both in Indonesia and in Indonesian‐occupied East Timor (Sissons, 1997; Smyth, 1991). There is, unsurprisingly, little acknowledgement of this coercive past in current reproductive health services.

Throughout my fieldwork, ideas about choice and voluntary participation in family planning and reproductive health were often framed—by local experts, health workers and community members alike—as “not being forced”. During the training program health care prior to independence was mentioned but there was no mention of the violence and oppression of the occupation, nor the accusations against the Indonesian family planning program. Rather, the specter of the Indonesian occupation moved in the background, mostly present in the repeated messages which denounced coercion, but never addressed coercions of the past.

In Timor‐Leste the discourse of choice in family planning is situated within a history of oppression, where choice does not only mean the right to choose, but also the right not to be coerced. Reproduction in this landscape is subject to multiple modes of “reproductive governance”—the mechanisms through which different groups of actors use encouragement, incitement or coercion that produce, monitor and control reproduction (Morgan & Roberts, 2012, p. 243), such as international NGOs, the Ministry of Health and the Catholic Church. For example, the facilitators delivering the training were invited by the National Institute of Health (INS) and included family planning specialists from INS as well as non‐profit health organizations including Marie Stopes International (MSI), Jon Snow Inc. (JSI), and the Timor‐Lorosa'e Centre for Natural Family Planning and Billings Ovulation Method—a Catholic organization.

The training was instigated and financed by the Korea International Cooperation Agency through their “Save Mother and Child” program (T, *Salva Inan no Oan*), targeting maternal and infant mortality. The training was a last‐minute addition to the program to use up remaining funds and participants received $10 per day as well as meals and accommodation. The 10‐day course included several modules, exams, and a practical element.

Altogether, 12 doctors, nurses and midwives participated. They had studied overseas in Indonesia and Cuba, as well as at the national university. WHO and UNFPA provided presentations and training manuals, translated from English to Tetum. Language was a challenge throughout as the Tetum translations were sometimes confusing, and many participants had studied in Indonesian or Spanish. The group was mostly females (25–50 years) from local families in the community aside from those on placements from other areas. Their profession gave them a high status particularly in rural villages. This class dynamic sometimes appeared in judgmental and condescending comments towards patients, but paternalism was also embedded with the training materials.

The first module of the training course covered the benefits of family planning. The facilitator, Helena, gave a WHO definition:

“Family planning allows individuals and couples to anticipate and attain their desired number of children and the spacing and timing of their births. It is achieved through use of contraceptive methods and the treatment of involuntary infertility”.

There was some confusion with this description. Whilst people used the Indonesian acronym KB to refer to contraceptives, the Tetum term “*Planeamentu familiar*” was more widely associated with planning financially or logistically for a family, including securing income and a place to live. Whilst this reflects the definition of family planning provided in the National Family Planning Policy (2004), in professional health settings, “family planning” was still most often a shorthand for contraception with fertility treatments rarely addressed. “Birth spacing”, allowing a 3‐year gap between pregnancies, was promoted to reduce maternal mortality, but it also implied that women would have fewer children overall. The *purpose* of family planning is to give people a choice, but the use of contraceptive methods is the desired *outcome*. The measurable targets of family planning programs in the global south tend to be contraceptive use (Hendrixson, 2019). Fertility reduction aims are implicit in such programs show how despite a language of individual choice, methods and goals are based on population control approaches (Brunson, 2020; Hendrixson, 2019; Nandagiri, 2021). In Timor‐Leste in 2018, a reproductive health organization advertised a vacancy for a marketing manager, to “increase” contraceptive use in the country. Whilst the discourse is one of individual choice, the end targets are increasingly used to fill “unmet need”, a category calculated from women who do not want more children and are not using contraceptives, assuming contraceptive use is always desirable (Murphy, 2017; Sasser, 2018).

The importance of freedom from coercion outlined on the first day of this family planning training course is not surprising considering Timor‐Leste's history of occupation and oppression, and allegations of genocide carried out by the Indonesian military. However, whilst negative and threatening language was seen as coercive, encouragements professing the benefits of family planning were not seen as unduly persuasive, but rather as providing a choice.

Narratives of “empowering” women proliferate in global family planning program with family planning represented as a “ubiquitous good” which brings both female empowerment and societal development (Nandagiri, 2021, S225). However, as a wealth of reproductive studies have shown, family planning programs past and present have brought both empowerment and disempowerment, and in both cases quite often place judgement and/or responsibility on women (Krause & Zordo, 2012). It is precisely the benevolent construction of choice which masks the inherent inequalities people face in exercising choice and autonomy over their reproductive lives. It is also this construction of choice, as freedom from coercion, that conceals the nuances of both concepts.

Scholars have argued the binary of coercion with choice is unhelpful, as this binary relationship doesn't accurately provide the full picture. Senderowicz argues that true free and informed consent sits on a spectrum which is affected by everyday limitations to choice as well as by covert manipulations (2019). The very positioning of choice in relation to coercion means that choices, unlike coercion, are unconstrained. This creates a dichotomous situation in which the client chooses to use family planning methods or doesn't.

In Timor‐Leste, reproductive choice, which emphasizes freedom from coercion, is discussed without addressing the historical coercion or the influence of Catholic Church. While the Indonesian occupation's history is recognized nationally, the influence on coercion and health care is shrouded in silence. The discourse of choice obscures past coercion and also conceals current coercive structures, paradoxically impeding the choices it aims to support.

## CHOICE CONCEALS COERCION

**FIGURE 1:**
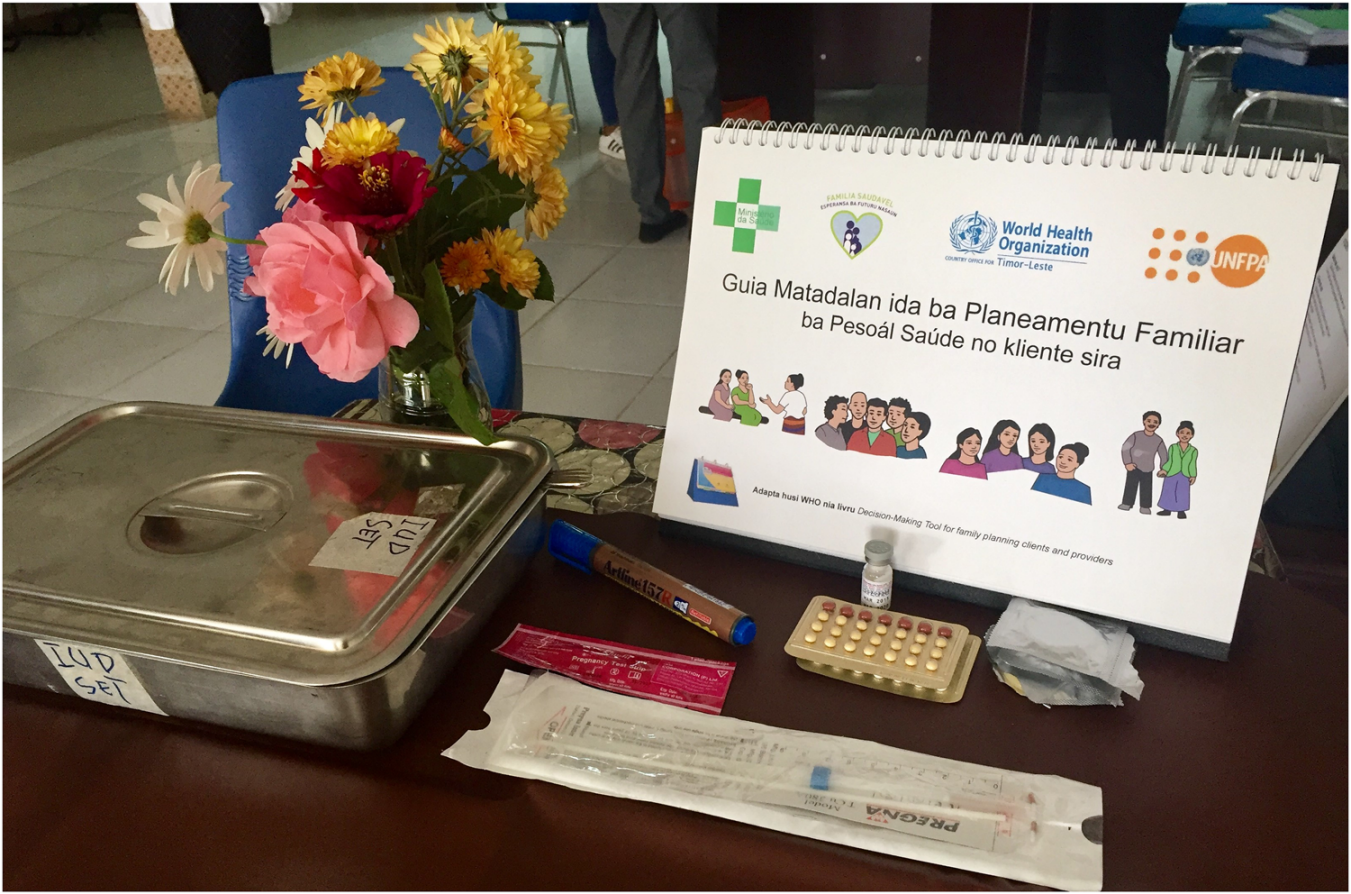
A desk for training participants to practice counselling their clients. On top is an IUD insertion set, a copper IUD, pregnancy test, contraceptive pills, condoms and a Depo Provera shot with a family planning guide for health staff and clients. Photo by Author. [This figure appears in color in the online issue]

On day two of the training, we reviewed the previous day's material:

“It's *always* the client that chooses, based on the information you give them”, Helena said firmly. However, the limitations of clients’ choice began to show as we split up to role play counselling sessions. This involved splitting in to pairs at prepared consultation tables (Figure 1). One person acted as the client whilst the other as the health professional, advised them using the materials and skills we had learnt so far. We fumbled through flip charts and a laminated booklet to navigate family planning methods: condoms, implant, copper Intrauterine Device (IUD), progesterone and combined hormonal pill, Depo‐Provera injection (Depo), tubectomy or vasectomy, the Billings method and breastfeeding. The charts were designed so the page facing the clients showed simple bullet points. On the back page, hidden from the client, was more detailed information for the practitioner. A booklet of flowcharts also directed the health practitioner through a series of YES or NO boxes to determine a client's eligibility for a particular method.

A male nurse questioned how they would get through all this material with each client. Outreach visits might mean treating over 100 people a day. Helena suggested starting with the clients preferred method to save time. As we struggled with the materials, the facilitators reiterated that clients made their own choices based on our guidance. This placed significant responsibility on healthcare providers to explain options clearly and assess safety. Ultimately, client decisions depended not just on their preferences but on the eligibility flow charts and the practitioner's expertise in using these tools. This highlighted a tension between promoting informed choice, following protocols, and tools developed without considering local dynamics.

During this session, a strikingly obvious factor affecting a client's choice of method emerged. One nurse, Lydia, described an event four months earlier:

“A patient said to me “Senhora, I want the injection”, but we had no Depo in stock. Our IUDs had expired, the implants had also expired, and all the pills had also expired, so everything was out of stock…she was really worried about getting pregnant again, and her youngest child was 10 months old.”

The facilitators were silent for a moment and exchanged a knowing look. They were all too familiar with this problem. ‘You should coordinate with other clinics so the patient can get Depo’, one advised.

“Sometimes they are also out of stock”, murmured someone next to me. In one interview, a senior midwife at the clinic showed me months of blank records from 2018 where contraceptives had been completely out of stock.^7^ Lack of availability was the one basic level at which a patient's choice was undermined, but there were other contradictions too.

“They keep saying it's up to the patient to decide, *always*,’ Natalia, a young nurse, complained on our walk home, ‘but what if they pick something that isn't right for them?”

Her colleague, Crista, made a face and responded rhetorically “If a patient asks for medicine that is going to kill them…do we give them medicine to kill themselves?”

This highlighted the conflict at the heart of the first few days’ trainings. The facilitators and training materials reinforced that choice was in the client's hands. Yet it was up to the doctor to tell the client what was best. If a client asked for a contraceptive method that conflicted with an existing medical condition, or if it was unavailable, the health worker should find an alternative. Importantly, we were taught this was also the case if the husband was not in agreement with the woman's choice’. Whilst in large part choice is negated by inequalities of access, choice also interacts with other discourses. Mol describes how a logic of choice perpetuates an ideal where care is turned into private decisions, foregoing discussions (Mol, 2008). Where patients are deemed ‘clients’ the language of choice carries more weight as patients are transformed into consumers.

Choice can be constrained by coercive factors like limited access, spousal disagreement, and domestic violence, making it a duty for medical professionals to offer alternatives. Reproductive coercion stems from both structural and interpersonal issues, which can hinder contraceptive autonomy even among those with good intentions. Senderowicz advocates for demographers to adopt a new metric for family planning that reflects women's autonomy in contraceptive choices (2019). However, measuring autonomy is complex due to various interrelated factors, including access to resources and legal or religious restrictions.

“Some people come and ask for the injection, and they hide their pregnancy,” said Lydia, “if you give them a pee pot, they fill it with water or ask someone else to pee in it.”

“But you can't do a urine test for every client” Helena said, “otherwise you're going to run out of the pregnancy tests very fast”.

“We do run out, but if we ask them if they are pregnant and they lie, then we give them some contraceptives, we could get in trouble.” complained Lydia.

Not only did the heavy assertion of patient choice complicate the medical professionals’ duty of care, but the issue of pregnant women requesting contraception also showed how the medical staff felt the need to protect themselves from being criminalized for causing harm. Helena tried to reassure the participants Depo would not cause miscarriage.

This illustrates a severe constraint on patients’ reproductive freedom in Timor‐Leste: the criminalization of abortion.^8^ Despite examples of women actively seeking abortion, abortion access was not addressed at all during the training. In 2018 public debates about infanticide and child abandonment were widespread and largely blamed on teenage pregnancy (Burke, 2022b). During these public debates access to abortion was never addressed and remained as a form of legislative coercion that leads to the concealment of pregnancies, infant abandonment and unsafe abortion.

Striving for choice is surely something to aim for when it comes to contraceptives, but the limitations on using contraceptive drugs, as well as their availability, show how the language of “choice” gives a false perception of reality in Timor‐Leste and elsewhere. A language of choice and access to some forms of contraception can coexist with the most coercive barrier to bodily autonomy. Choice exists alongside a range of structural inequalities that might be minimally coercive, yet it also sits alongside criminalization of abortion.

What is perceived as choice is constrained by factors outside of the person's control, from access and availability, to eligibility, social relations and legal constraints. These sorts of inequalities, caused by varying modes of reproductive governance, are what reproductive justice proponents might refer to as “environmental” or “community” conditions and extend to racism, discrimination and other intersecting inequalities. There was little room for this in the training material. Much of it assumed a rational actor making a “prior‐informed choice” but left little guidance about what to do in spaces of uncertainty or when actors were not acting towards a particular imagined future. In demography and global health, reproductive intentions are presented as static and rationalized. But in an environment without access to abortion, a woman might seek a Depo injection in the hope it will induce an abortion.

The illusion of choice is quickly shattered in a local context where access to healthcare is unequal, and when reproduction isn't static, individual or biomedical. Societal expectations and personal relationships add another layer of complication. Privacy was a significant concern, as clinics and small health posts often lacked private spaces for consultations, and people frequently interrupted. Unmarried people avoided seeking contraception unless they were sure of anonymity, and women risked exposure to their husbands or community if seen visiting family planning clinics.

In 2017, a draft revision of the National Family Planning Policy sparked controversy for describing users as “married” couples (*kaben nain*). Leaked to activists before public consultation, it was criticized for restricting contraceptive access for younger, unmarried people, reflecting Church teachings on abstinence. The draft policy was not addressed in the training, and Helena loudly reminded us under current policy ‘ANYONE’ could receive family planning, married or unmarried. Despite this, many of the participants told me they would not give contraceptives to unmarried people, particularly if they were young and still at school or university. During roleplays, participants acting as clients used simple scenarios like, “I need the pill because I have too many children” or “My husband supports contraception.” When I role played an unmarried client, most hesitated nervously, unsure how to proceed.

When I asked about giving contraceptives to young people Lydia admitted, ‘I'd tell them to focus on their studies first and tell them to go back to school…they are still young, they would be too scared to come and ask for it anyway, someone might see them’.

“Why would they need it?” One nurse responded.

Many participants also supported requiring husbands’ permission for married women to use family planning. The training encouraged involving husbands in consultations to ensure their agreement and understanding, aiming to reduce domestic violence and conflicts, including aggression toward medical staff, which had occurred before. The 2017 draft family planning policy was already unofficially in place, as health workers were unlikely to give contraceptives to young unmarried people, and encouraged consent of husbands. In 2017 *Grupu Feminista* and other civil society actors had succeeded in raising awareness, and the revised policy was shelved. In 2022 however the revised national family policy was passed without further public consultation, meaning access for young or unmarried people remains a challenge.

Despite reinforcing that the client had the right to decide what she wanted to use, the training material and the challenges posed by the participants acknowledged the decision to use family planning services was not solely the choice of the individual, but it confronted issues of eligibility and availability and was entangled in a web of wider relationships. It is these quotidian, normalized forms of coercion—such as hidden information, limited time, stock levels, restrictive laws and policies and moral judgements—“distort the process of consent and result in forcing a person to do something against their will” (Boydell et al., 2023, 11).

Coercion and choice, in their multiple definitions, exist in a diversity of ways at the same time. It is only through theorizing the relationship between the two that we get a full picture: choice is not the antithesis of coercion, rather they overlay one another. In this space, choice and coercion coalesce—the space in which one language can be used to mask or conceal the other. Yet there is another twist in this relationship: it is not only that the liberating language of choice conceals hidden forms of coercion, but also, as I show next, a coercive practice can paradoxically reveal a hidden choice.

## COERCION REVEALS A HIDDEN CHOICE

Subverting the main narrative of choice and the discourse of reproductive rights, one module of the training brought forward a different narrative. Flàvio, led the module on natural methods. He worked for the “Centre for Natural Family Planning and Billings Ovulation Method”, a health center on the grounds of the Cathedral of the Immaculate Conception.

“The woman is like the land”, Flàvio said, “…she receives. If you plant cassava, you get cassava…if you plant bananas and get tomatoes, uh‐oh, something's wrong!” Flàvio's joke suggested infidelity. His crass approach to teaching about “natural” methods was sexist and dogmatic. It began with the assumption that people acted on “natural” impulses and that God, and the body God have given them, were in control. Flavio deviated from the WHO training slides and used his own to dispel some “myths”. The Billings method was not a “traditional” method, but a “body” method. It was scientific, he said, but also *natural*: Billings was not any less “modern” than other methods, and it was free of chemicals and artificial materials.

Flavio played on health concerns associated with unnatural chemicals. He backed up his claims with some biological mathematics:

“A woman has approximately 400,000 eggs. Only 400–500 of these eggs will be released from the ovaries during a woman's lifetime; the eggs that aren't released by the woman are given back to God…” He picked on Rita:

“How old are you?”

“31,” Rita answered timidly.

“When was your first period?” he asked.

“16 years old,” she reddened.

Flavio formulated a sum: “So if you release an average of 12 eggs a year over the last 16 years, so far you have released 192 eggs….so that means you've got 208 eggs left,” he announced to the room. All female participants exchanged looks. Many of us were a similar age to Rita, and according to Flavio we had approximately half of our “God‐given” eggs left.

With these calculations Flavio reinforced the natural authority of the body, pointing out that time was *not* a choice nature was giving us.

“Today we are going to learn about the woman's body. Why? Because it's always the women that face the problems…. a man is ready 24 h a day… We men are the *huun* (origin), you women make the children, but we men came from the beginning, you came from the rib, this is reality…”

The Billings method was widely promoted in Timor‐Leste by both the Church and family planning programs alike. We labored over our Billings charts, with numbers and symbols, and struggled to describe different viscosities of vaginal discharge. Flavio told us we found it difficult because we didn't understand it properly yet. He did not advocate *free* choice; rather, he told us making a good choice was based *on knowledge and awareness*, which he saw as predetermined by nature, made by God.

Interestingly, he promoted Billings as the *better* method because it gave people more *options*. It could help you *have* children as well as stop having them and was even a way to plan for a boy or girl. Flavio was using the attraction of choice to conceal his bias, but he didn't hide that choice was limited by wider structures:

Do you know how much money the Ministry of Health spends on contraceptives? Not a cent. It comes from the agencies, UNFPA, how much do they give? $200,000 USD. The agencies see Timor and think, oh Timor needs help, let's help…So when UNFPA stops buying Depo[Provera], the medicine, where is it going to come from? Are you going to go to the sea and collect water? …earlier Lydia talked about not having well managed stock, but one alternative is natural methods. Dr. Billings wasn't creating anything new, no medicine or contraceptives, he was working with what God had given us.’

Flavio's stance embodies the attitude that a choice discourse was developed to counter. For all his crassness and sexism, he acknowledged structural coercions at a political scale. It is precisely the discourse of choice that obscures, disguises, alludes and assumes equality across different reproductive environments, here Flavio was making some of that inequality transparent.

I walked home with two young midwives — Lili and Natalia, who complained about Flavio's crassness.

‘His position is very clear though; he supports Billings all the way.’ Natalia laughed. His apparent honesty earned him some respect.

‘I think Billings is the best,’ Lili said, ‘it fits in best with our culture and religion, *and* it helps you get pregnant*’*.

After class, three female participants had stayed behind to ask Flavio's advice on conceiving. One was Mia, a good friend I watched soap operas with. Three months after the training she told me ‘Sister! I'm pregnant, I used Billings for just three months, and it worked!’ She was overjoyed.

Crass and judgmental as he was, Flavio never asserted that “choice” was in people's hands, in a scenario where the supply of biomedical contraceptives was reliant on international aid and development funding, and where supplies often ran low. By acknowledging that reproductive choice was not only down to individuals, playing on pro‐natal desires, obligations, and Catholicism, as well as stating his opinion openly, Flavio gained trust. Whilst global health family planning discourses are shaped by an anti‐natal bias (Bhatia et al, 2020), Flavio's pro‐natal stance supported an option most people in Maubisse desired: having children (Burke, 2022a). Munro and Widmer (2022) describe how assisted reproduction has been a topic of much of reproductive studies literature, but for many populations, it is a case of abandonment rather than assistance. Yet many communities in under resourced areas lack health care technologies care that enable and support pregnancy as well as those that prevent it.

By not participating in a discourse of choice, one challenged by a myriad of social and structural issues, Flavio's sexist and dogmatic views, were seen as genuine. Ironically, the way Flavio promoted Billings as a tool for fertility awareness opened desirable choices: getting pregnant and supposedly determining the sex of a child (families in Maubisse favored an equal number of boys and girls). Ironically, Flavio's paternalistic sexism, clear bias and moral inducements revealed a choice that was not addressed by the other biomedical family planning options provided. It also highlights how the discourse around choice in family planning can be contradictory, leading to confusion and lack of trust in biomedical interventions. However, Flavio's seeming transparency also opened avenues to sow distrust: on the final day of his training, he circulated leaflets from an Australian pro‐life organization which included a message claiming that IUDs and hormonal contraceptives cause cancer.

## COERCION AND CHOICE AS IATROGENESIS

Iatrogenesis has been recently revisited by medical anthropologists. Varley and Varma (2021) advocate for a broader understanding of medicine's ability to cause harm, one which captures not only clinical processes but also the far‐reaching and lingering effects beyond the clinic. The concealed coexistence of choice and coercion in discourse and practice is part of this broader iatrogenic scope. Through concealment — lecturing about choice whilst failing to address coercive pasts and presents, failing to offer reproductive assistance, or only offering through a religious lens — we reproduce iatrogenic harm in family planning.

Boydell et al (2023) illustrate different degrees along a continuum of coercive practices to document the different forms of coercion found in long‐acting forms of contraception (LARCs). However, the authors caution that such a continuum lacks nuance, particularly due to changes over time in what are considered coercive practices. Viewing coercion and choice in a coexisting relationship characterized by concealment shows the dynamics in which they change and obscure one another. The coexistence of choice and coercion is concealed in the silences about past coercion and the conditions in which choice became a central narrative in family planning discourse. In Timor‐Leste, choice is presented as freedom from coercion, without any acknowledgement of where that coercion might have come from, or what form it may have taken. As the family planning training course showed, a discourse of choice obscures forms of coercion, both structural and interpersonal, that people might encounter — from the quotidian to the criminalization of abortion. Such concealment also leads to people themselves becoming agents of concealment in the process, e.g., to access medicines they hope might induce abortion.

Kligman (1998) identifies duplicity and complicity as forms of behavior that contribute to reproductive violence in authoritarian Romania. Whilst the law seeks to locate responsibility for coercion in individuals or institutions (Boydell & Smith, 2023), I am not theorizing this relationship as intentionally duplicitous. Whilst duplicitous actions and complicity may be present in some of what I present, I conceptualize concealment not as a deliberate act but as something which often arises as an unintended consequence. Whilst no doubt there are those who seek to limit reproductive freedom, the unstable and concealed coexistence of coercion and choice, is one of the unintended consequences of the wider politics of reproduction and population. By outlining both choice and coercion and their many forms and practices, and asking women what they want, we might be unsurprised when women choose a range of options based on dynamic social and environmental contexts. At the Lauhili clinic, and in the wider Maubisse area, where I conducted my research, female health professionals were quick to choose fertility awareness options, *alongside* access to contraceptives, *as well as* better access to maternal and infant care, education and a safe environment in which to raise their children (Burke, 2022b).

An ideal of choice masks the social and structural inequalities that limit free choice and are considered forms of coercion, for example, limiting access, limited information, omissions, persuasion and finally direct abuse and harm. These forms of coercion did not occur because choice as a concept wasn't well explained or understood, but because it *was*. Acting under a cloak of choice, particularly when choice appeared to be rife with contradictions, enabled coercion to take place. The client consented to attend a consultation on family planning. Rita's superior pressured her to persuade the client to get an IUD, and Rita presented a range of choices. Rita omitted details and used persuasive language, the husband chose the IUD, the client consented, and the insertion of the IUD was rushed and borderline abusive. The example oscillates back and forth between coercion and choice, moments of consent and moments of persuasion and pressure.

Choice and coercion coexist at multiple scales throughout family planning processes, overlaying and obscuring each other. More overtly coercive approaches that restrict some choices can also reveal others which were previously hidden, such as fertility awareness and the antenatal focus of family planning, with its primary objective to prevent pregnancy rather than equally prevent or assist it. It is this relationship of coexistence, characterized by concealment, that I argue leads to iatrogenic harm in family planning. This is both a call for transparency and acknowledgement of the unequal conditions reproductive justice proponents have sought to address. This requires us to be clear about how coercion coexists alongside choice in practice.

## ENDNOTES

1 Names and places have been anonymized to protect participants.

2 Having a monthly period is desirable at these clinics as it is seen as a sign of healthy fertility.

3 Anonymized.

4 Ethical approval came from Timor‐Leste's National Health Institute *Instituto Nacional de Saude* (INS).

5 The training was conducted in Tetum, the national language of Timor‐Leste.

6 See Women in Conflict/National Public Hearing (2005).

7 The provision of contraceptives was a problem family planning stakeholders were aware of and were working to address in 2019.

8 Only to be done in the case of danger to the mother's life, with the authorization of three doctors (one trained in OB GYN, and a fourth doctor to carry out the procedure. See Belton et al. 2009
